# Phase 2 trial of everolimus and carboplatin combination in patients with triple negative metastatic breast cancer

**DOI:** 10.1186/bcr3634

**Published:** 2014-03-31

**Authors:** Jasmeet Chadha Singh, Yelena Novik, Stacey Stein, Matthew Volm, Marlene Meyers, Julia Smith, Coral Omene, James Speyer, Robert Schneider, Komal Jhaveri, Silvia Formenti, Victoria Kyriakou, Benson Joseph, Judith D Goldberg, Xiaochun Li, Sylvia Adams, Amy Tiersten

**Affiliations:** 1 Department of Medicine, Division of Hematology-Oncology, New York University Medical Center, New York, USA; 2 New York University Cancer institute, New York, USA; 3 Department of Medicine, Division of Hematology-Oncology, Yale Medical Center, New York, USA; 4 Department of Radiation Oncology, New York University Medical Center, New York, USA; 5 Clinical trials office, NYU Cancer Institute, New York, USA; 6 Division of Biostatistics, New York University School of Medicine, New York, USA; 7 Department of Medicine, Division of Hematology-Oncology, Mt Sinai School of Medicine, Mt Sinai School of Medicine, 1176 Fifth Avenue, New York, NY 10029, USA

## Abstract

**Introduction:**

Rapamycin acts synergistically with platinum agents to induce apoptosis and inhibit proliferation in breast cancer cell lines. Combination of everolimus also known as RAD001 (oral mammalian target of rapamycin (mTOR) inhibitor) and carboplatin may have activity in metastatic triple-negative breast cancer (TNBC).

**Methods:**

The primary objective of this study was to determine clinical benefit rate (CBR), that is (complete remission (CR) + partial remission (PR) + stable disease (SD) lasting ≥6 months) and the toxicity of everolimus/carboplatin in women with metastatic TNBC. Prior carboplatin was allowed. Treatment consisted of intravenous carboplatin area under the curve (AUC) 6 (later decreased to AUC 5 and subsequently to AUC 4) every 3 weeks with daily 5 mg everolimus.

**Results:**

We enrolled 25 patients in this study. Median age was 58 years. There were one CR, six PRs, seven SDs and eight PDs (progression of disease). CBR was 36% (95% confidence interval (CI) 21.1 to 57.4%). One SD was achieved in a patient progressing on single agent carboplatin. The median progression free survival (PFS) was 3 months (95% CI 1.6 to 4.6 months) and overall survival (OS) was 16.6 months (95% CI 7.3 months to not reached). There were seven patients (28%) with ≥ grade 3 thrombocytopenia; three (12%) with grade 3 neutropenia (no bleeding/febrile neutropenia) and one (4%) with grade 3 anemia. Greater hematological toxicity was seen in the first seven patients treated with carboplatin AUC5/6. After the amendment for starting dose of carboplatin to AUC 4, the regimen was well tolerated with only one out of 18 patients with grade 3 neutropenia and two patients with grade 3 thrombocytopenia. There was only one case of mucositis.

**Conclusion:**

Everolimus-carboplatin was efficacious in metastatic TNBC. Dose limiting hematological toxicity was observed when AUC5/6 of carboplatin was combined with everolimus. However, carboplatin AUC 4 was well tolerated in combination with everolimus with continuing responses.

**Trial registrations:**

ClinicalTrials.gov NCT01127763.

## Introduction

Triple negative breast cancer (TNBC) is a breast cancer subtype characterized by the lack of expression of estrogen receptor (ER), progesterone receptor (PR) as well as HER-2 neu amplification (grade 3+ on immunohistochemistry (IHC) or fluorescent in-situ hybridization (FISH)-positive) on the cancer cells and constitutes about 15% of all breast cancers [1]. Women with TNBC tend to be younger and demonstrate early recurrence (within the first 2 years), higher histological grade, higher rate of visceral metastasis and increased mortality rates when compared to hormone-positive breast cancer [2]. Prognosis for metastatic TNBC is especially poor with median survival of only one year when compared to about 2 · 3 years with other subtypes of breast cancer [3].

Due to lack of targeted therapies, there is no standard treatment of choice for triple-negative breast cancer and chemotherapy remains the accepted standard. Many chemotherapeutic agents have been reported to have clinical activity either as single agents or in combination. Seventy percent of breast cancers with breast cancer (BRCA)-1 germline mutations are triple negative, which suggests a shared carcinogenic pathway between the two [4,5]. Both TNBC and BRCA-1-associated breast cancers are particularly sensitive to DNA crosslinking agents such as platinum compounds, due to defective DNA repair by homologous recombination [6,7]. Lehmann *et al*. used gene expression profiles to identify six TNBC subtypes with distinct gene expressions and ontologies; each responsive to unique therapeutic agents *in vitro* and in xenograft models. BRCA-1 mutant and non-BRCA mutant basal-like subtypes (BL-1 and BL-2) expressed high levels of genes involved in cell proliferation and DNA damage response. The representative cell lines of these subtypes were highly sensitive to cisplatin [8]. In a phase 2 study of cisplatin in BRCA-1-mutated metastatic triple-negative breast cancer, overall response rates as high as 80% (complete response (CR) rates of 45% and PRes 35%) have been observed. Median time to progression was 12 months: 55% of patients had undergone prior chemotherapy for metastatic disease [9]. Platinum agent combinations with other chemotherapeutic agents were evaluated in the metastatic setting in a retrospective study. Of the patients, 63.5% were triple negative. The most common combination was Cisplatin and ifosfamide, used in 70% of the subjects. Other chemotherapy combinations used were carboplatin-ifosfamide (5.6%); cisplatin-ifosfamide-bevacizumab (4.2%); cisplatin-gemcitabine (2.8%); cisplatin-docetaxel (4.2%); cisplatin-cyclophosphamide (2.1%) and navelbine platine ifosfamide (7.7%). Response rates to individual regimens were not reported but the response rate to the platinum-based regimens in TNBC was 33.3% versus 22.0% in the non-TNBC group [10]. In another phase II study, the combination of carboplatin with gemcitabine for metastatic TNBC showed an overall response rate (ORR) of 32% [11].

Everolimus is a selective inhibitor of mammalian target of Rapamycin (mTOR). The phosphotidyl-inositol 3 kinase (PI3K)/AKT/mTOR pathway is known to be dysregulated in a wide spectrum of human cancers including breast cancer [12]. mTOR acts via the PI3K-AKT signaling pathway, leading to phosphorylation of targets including p70-S6 Kinase 1 (S6K1) and eukaryotic initiation factor 4E binding protein 1 (4E-BP1). Through this pathway, mTOR plays a key role in the regulation of many cellular processes, including cell proliferation, survival, and apoptosis [13].

Loss of phosphatase and tensin homolog (PTEN) expression has been reported in 37 to 74% of metastatic triple-negative breast cancers [14-16], which results in activation of the PI3K/PTEN/AKT/mTOR pathway, making mTOR inhibitors also a potential molecular target for treating TNBC [17,18]. Activation of the PI3K/AKT/mTOR pathway in breast cancer portends a worse prognosis, increased aggressiveness and resistance to treatment [19].

Everolimus has a potential to act directly on the tumor cells by inhibiting tumor cell growth and proliferation as well as indirectly by inhibiting angiogenesis, leading to reduced tumor vascularity (via potent inhibition of tumor cell hypoxia induced factor-1 (HIF-1) activity, vascular endothelial growth factor (VEGF) production and VEGF-induced proliferation of endothelial cells) [20,21].

There is also preclinical evidence suggesting that everolimus enhances the sensitivity of breast cancer cell lines to carboplatin resulting in synergistic inhibition of cell proliferation and caspase-independent apoptosis [22]. Mesenchymal-like TNBC subtype was found to be extremely sensitive to dual PI3K/mTOR inhibition in preclinical models [8]. In other preclinical studies, everolimus was found to have efficacy against basal-like subtypes of TNBC especially if they expressed endothelial-derived growth factor (EGFR) receptor or cytokeratin (CK) 5/6 [23].

Everolimus has been evaluated in hormone-positive and Her-2 positive MBC with promising results. In the phase 3 BOLERO-2 trial, 724 patients with hormone-positive metastatic breast cancer (MBC) were treated with exemestane alone versus exemestane plus everolimus. Progression-free survival (PFS) was 10.6 months in the combination group versus 4.1 months in the exemestane alone group (hazard ratio (HR) 0.36; 95% CI 0.27, 0.47; *P* <0.001). Response rates were 0.4% and 9.5% respectively [24]. In preclinical models, mTOR inhibitors synergize with trastuzumab and have shown to cause complete regression of mouse Her-2-positive mammary tumors [25]. In a phase I/II trial of trastuzumab combined with mTOR inhibitor everolimus for Her-2-positive MBC, partial responses were seen in 15% of patients and stable disease lasting for at least 6 months in 19% (clinical benefit rate (CBR) 34%) [26]. BOLERO-3 is a phase III trial comparing vinorelbine and trastuzumab alone or in combination with everolimus in 569 patients with metastatic Her-2-positive breast cancer resistant to trastuzumab. The preliminary findings of this study were presented in the American Society of Clinical Oncology annual meeting in 2013. The median time to progression was 5.8 months in the control arm and 7 months in the everolimus arm (HR for progression 0.78; 95% CI 0.65, 0.95; *P* <0.01). The response rates were not significantly different between the two groups (41% in the everolimus arm versus 37.2% in the non-everolimus arm). The data on overall survival, the secondary endpoint of this study, are not yet mature [27].

The combination of everolimus with carboplatin has been tested for non-triple-negative MBC (12 patients were hormone receptor-positive and 5 were Her-2-overexpressing) in a phase 1 dose-determination study. In this study 15 patients with pretreated MBC were treated with weekly carboplatin, with an area under the curve (AUC) of 2, and different dose levels of everolimus (level I: 2.5 mg, level II: 5 mg, level III: 7.5 mg and level IV: 10 mg). Three patients were each treated with levels I through III and six were treated with level IV. Three patients had PRes and three had stable disease (SD) lasting >24 weeks, making the CBR 40%. Median PFS was 19 weeks and overall survival (OS) was 35.3 weeks [28]. This is the first study that looks at an mTOR inhibitor in combination with a platinum agent in triple-negative breast cancer.

## Methods

This was a single-institution phase II trial. The primary objective of the study was to determine the clinical benefit (CR + PRes + SD lasting ≥6 months) and toxicity of everolimus and carboplatin combination in women with metastatic TNBC who have had 0 to 3 prior chemotherapy regimens for metastatic disease.

The institutional review board (IRB) of the New York University provided ethical approval for this study. All patients were required to sign IRB-approved informed consent in order to be eligible for the study. All the enrolled patients were treated in compliance with the Declaration of Helsinki. Eligible patients were women with metastatic histologically confirmed triple-negative breast cancer (ER <10%, PR <10%, HER-2neu IHC 0 or 1, or FISH-negative). Additional eligibility criteria included age ≥18 years; World Health Organization (WHO) performance status ≤2; adequate bone marrow function (absolute neutrophil count ≥1 · 5 × 10^9^/L, platelets ≥75 × 10^9^/L, hemoglobin >9 g/dL); adequate liver function (serum bilirubin ≤1 · 5 × upper limit of normal (ULN), international normalized ratio (INR) ≤1 · 5 for patients not on warfarin and INR ≤3.0 for patients on warfarin, alanine transaminase (ALT) and aspartate transaminase (AST) ≤2 · 5 × ULN (≤5 × ULN in patients with liver metastases)); patients on stable dose of lower molecular weight heparin for >2 weeks at time of treatment; adequate renal function (serum creatinine ≤1 · 5 × ULN); fasting serum cholesterol ≤300 mg/dL or ≤7 · 75 mmol/L and fasting triglycerides ≤2 · 5 × ULN (in case one or both of these thresholds were exceeded, the patient could only be included after initiation of appropriate lipid-lowering medication); baseline lung computed tomography (CT) scan or positron emission tomography (PET)/CT; oxygen saturation ≥90% in room air; and negative serum pregnancy test within 7 days prior to starting treatment. Patients could have had 0 to 3 prior regimens for metastatic disease and prior bevacizumab-treated patients were eligible. Exclusion criteria included: history of having received other anticancer therapies or investigational drugs within 2 weeks of the start of the study drug; major surgery or significant traumatic injury within 4 weeks; patients receiving chronic systemic corticosteroids at ≥20 mg/day or other immunosuppressive agents; history of immunization with attenuated live vaccines within one week of study entry; uncontrolled brain or leptomeningeal metastases; other malignancies within the past 3 years except for adequately treated carcinoma of the cervix or basal or squamous cell carcinomas of the skin; uncontrolled medical conditions such as symptomatic congestive heart failure of New York Heart Association Class III or IV, unstable angina pectoris, symptomatic congestive heart failure, myocardial infarction within 6 months of start of the study drug; serious uncontrolled cardiac arrhythmia or any other clinically significant cardiac disease; severely impaired lung function (spirometry and diffusing capacity for carbon monoxide (DLCO) that is 50% of the normal predicted value and/or oxygen saturation that is 89% or lower at rest in room air); uncontrolled diabetes (fasting serum glucose ≥1.5 × ULN); uncontrolled severe infections; liver disease such as cirrhosis, chronic active hepatitis or chronic persistent hepatitis; known history of HIV seropositivity, impairment of gastrointestinal function or gastrointestinal disease that may significantly alter the absorption of everolimus (ulcerative disease, uncontrolled nausea, vomiting, diarrhea, malabsorption or small bowel resection); active, bleeding diathesis; pregnancy or lactation; prior treatment with an mTOR inhibitor or known hypersensitivity to mTOR inhibitors; history of noncompliance to medical regimens; unwillingness to comply or inability to comply with the protocol, or an ongoing alcohol or drug addiction.

### Baseline assessment

Patients were required to have baseline pulmonary function tests if their oxygen saturation was lower than 90% on room air. Either PET/CT or CT chest/abdomen/pelvis or bone scan were obtained at baseline and then after every two cycles of treatment (that is, every 6 weeks). At baseline, tumor lesions were categorized as measurable or non-measurable by the response evaluation criteria in solid tumors (RECIST) 1 · 0. a physical examination, vital signs, and tumor markers (carcinoembryonic acid (CEA) and cancer antigen (CA) 27/29) were checked at baseline and then every 3 weeks. A baseline electrocardiogram was also obtained. Laboratory tests included a complete blood count, prothrombin time (PT) (INR), blood chemistry and liver function tests, serum lipid profile and standard urinalysis dipstick assessment. All were screened for hepatitis B and hepatitis C infection.

### Treatment plan

According to the original study plan, carboplatin AUC 6 was to be given intravenously every 3 weeks: 5 mg of everolimus was to be given daily with a three-patient run-in and then 10 mg daily if there were no dose-limiting toxicities. Due to high incidence of severe thrombocytopenia seen with carboplatin AUC 6, the dose of carboplatin was first amended to AUC 5 on 2 August 2010 and then to AUC 4 with 5 mg of everolimus (with no escalation of everolimus to 10 mg) on 4 April 2011.

According to the most recent study plan, carboplatin was administered with AUC of 4 on day 1 of every cycle, and repeated every 3 weeks. Everolimus was provided by Novartis. The patients were instructed to take 5 mg of everolimus orally once daily continuously from study day 1 until progression of disease or unacceptable toxicity. Patients were instructed to take everolimus in the morning, at the same time each day.

### Statistical methods

**Figure 1:**
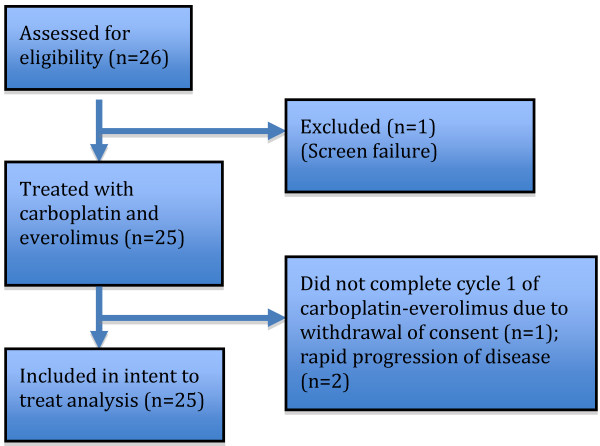
Consort figure.

This study was a single-stage phase II trial. With 25 subjects (Figure 1), this study had 80% power to test the null hypothesis that the clinical benefit rate (CR + PRes + SD ≥6 months) is ≤0 · 10 versus the alternative hypothesis that clinical benefit rate is ≥ 0 · 30. If the number of responses (including CR + PR + SD ≥6 months) was six or more, the hypothesis that P ≤0 · 10 would be rejected with a target alpha error rate of 0 · 050 and an actual error rate of 0 · 033. If the number of responses was five or less, the hypothesis that *P* ≥0 · 30 would be rejected with a target error rate of 0 · 200 (power of 80%) and an actual error rate of 0 · 193. These estimated clinical benefit rates are derived from benchmarks from prior studies in similar patient populations.

Descriptive statistics were used to summarize the patient demographic characteristics and adverse events. The benefit rate was estimated along with the exact 95% CI. Kaplan-Meier curves were provided for PFS and OS in this study; the corresponding median survival times and 95% CI were also estimated. SAS 9 · 3 was used for all analyses.

## Results

### Patient characteristics

Additional file 1: Table S1 shows the distributions of the demographic characteristics of the 25 patients: 44% of patients had received no prior line of treatment for metastatic disease, 28% had received one prior line of treatment, 24% had received two prior lines of treatment and 4% had received three prior lines of treatment for metastatic disease.

### Efficacy

In this study clinical benefit (CR + PRes + SD of >30% lasting more >6 months) was achieved in 9/25 patients (36%; 95% CI 21 · 1, 57 · 4%). There was one patient with CR, six with PRes, seven with SD and eight with PD. One patient with CR and four with PR were on a carboplatin dose of AUC 4 combined with 5 mg of everolimus. Two patients had stable disease lasting longer than 6 months. Three patients did not complete the first cycle of chemotherapy for the following reasons: withdrawal of consent in one patient; and rapid progression of disease in two other patients. SD was achieved in one patient progressing on single-agent carboplatin at study entry*.* Two patients had to be taken off the study due to treatment toxicity.

### Response by carboplatin dose

Among the four patients treated with carboplatin AUC 6 and everolimus 5 mg daily, there was one with PRes and one with SD lasting longer than 6 months. Among the three patients treated with carboplatin AUC 5, there was one with PR and one with SD lasting longer than 6 months. Among the 18 patients treated with AUC 4, there were one with CR and four with PRes (response rate (RR) and CBR 28%).

**Figure 2:**
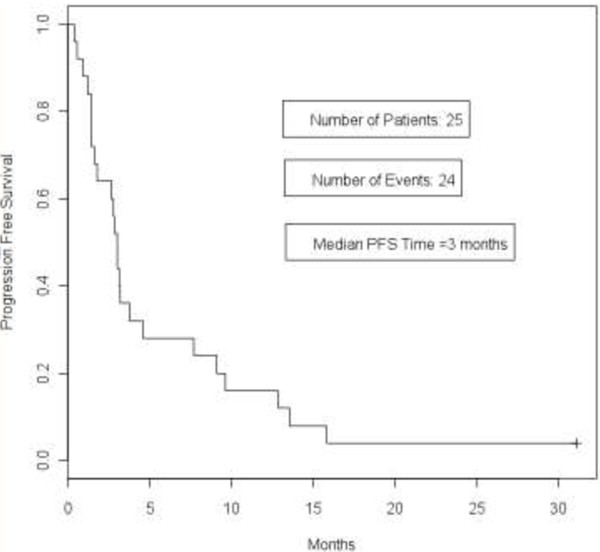
**Progression-free survival curve.** Kaplan-Meier curve showing progression-free survival in patients with metastatic triple-negative breast cancer who were treated with everolimus and carboplatin. The median PFS time was 3 months (95% CI 1 · 6, 4 · 6 months).

**Figure 3:**
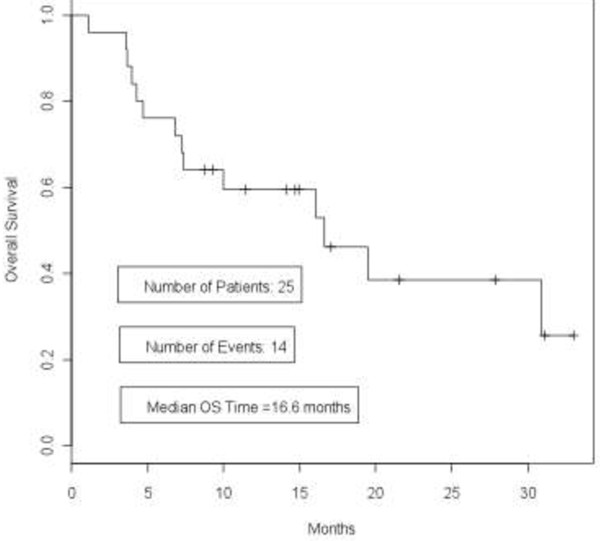
**Overall survival curve in patients with triple-negative breast cancer treated with everolimus and carboplatin.** Kaplan-Meier curve showing overall survival in patients with triple-negative breast cancer who were treated with everolimus and carboplatin. Median overall survival (OS) was 16 · 6 months (95% CI 7.3 months - not reached).

Kaplan-Meier curves for PFS and OS are shown in Figures 2 and 3 respectively. The median PFS time was 3 months (95% CI 1 · 6, 4 · 6 months) and overall survival time was 16 · 6 months (95% CI 7.3 months - not reached).

### Toxicity

Hematological toxicity (thrombocytopenia, neutropenia and anemia) was the most common high-grade toxicity observed especially in patients who were treated with higher doses of carboplatin (AUC 5 and 6). Hematological toxicity data are provided in Additional file 2: Table S2. Seven patients (28%) had grade 3 or higher thrombocytopenia. Five of these seven patients were treated with carboplatin doses AUC 5 or 6. Since protocol amendment, only two of the eighteen patients (11%) treated with carboplatin doses of AUC 4 had grade 3 thrombocytopenia. Three patients (12%) had grade 3 neutropenia with two of these patients being treated prior to second amendment for carboplatin dose. There was no febrile neutropenia, and no cases of severe bleeding. There was one case of grade 3 anemia. Growth factor support was required in seven patients (28%) and transfusion support in six patients (24%).

Non-hematological toxicity data related to the treatment are shown in Additional file 3: Table S3. This study is notable for minimal incidence of mucositis (one patient, 4%), that can probably be attributed to the lower dose of everolimus used. The other grade 3 non-hematological toxicities included dehydration (n = 1, carboplatin AUC 4), nausea and vomiting (n = 1, carboplatin AUC 4), and hypersensitivity to carboplatin (n = 1, carboplatin AUC 4). Grade 3 insomnia (n = 1), headache (n = 1), grade 3 cellulitis (n = 1), grade 3 elevation of INR in the setting of infection (n = 1); grade 3 foot infection (n = 1) and shortness of breath (n = 2) were also observed but these adverse events were thought to be unrelated to treatment. There have been no reports of interstitial lung disease secondary to everolimus in our study patients.

The four patients in the AUC-6 carboplatin cohort received 70% of the planned total dose of carboplatin, the three patients in the AUC-5 cohort received 68% of the planned dose and the 18 patients in the AUC-4 cohort received 64% of the total planned dose. This result seems paradoxical but can be attributed to the fact that in one patient in the AUC-4 group, carboplatin was discontinued after four cycles but the patient continued to be stable on single-agent everolimus for 14 more cycles. If we exclude that patient, 84% of the planned dose of carboplatin could be delivered in the AUC-4 group. Fifteen patients (60%) required treatment interruptions and nine (36%) patients required dose reductions. In seven patients, the dose of carboplatin was further decreased to AUC 3; in one patient carboplatin was discontinued and the patient had long-lasting partial response on single-agent everolimus. In five patients, the dose of everolimus was decreased to 5 mg on alternate days. Five patients (20%) were hospitalized for the following reasons: hypersensitivity reaction to carboplatin, cellulitis (unrelated to treatment regimen), foot infection (unrelated to treatment regimen), shortness of breath (unrelated to treatment regimen), headache and dehydration respectively.

## Discussion

We demonstrated the efficacy of the combination of everolimus with carboplatin in triple-negative metastatic breast cancer. Everolimus 5 mg daily given orally and carboplatin AUC 4 administered intravenously every 3 weeks was both safe and efficacious in metastatic TNBC with a CBR of 36% and median PFS of 3 months. When carboplatin was administered at a dose of AUC 5 or greater, higher incidence of grade 3 or higher hematological toxicity was observed. However, at the recommended dose of carboplatin at AUC 4 the combination regimen has been very well-tolerated and anti-tumor responses were observed.

Combination of carboplatin and cetuximab, an anti-EGFR agent, was recently studied in a phase II clinical trial by Carey *et al*. for metastatic TNBC. The study comprised two arms, arm 1 receiving cetuximab alone with carboplatin added on progression, and arm 2 receiving cetuximab plus carboplatin from the beginning. Of patients in arm 1, 32% and 52% of patients in the combination arm had received no prior chemotherapy for metastatic disease. The response rate to single-agent cetuximab was 6% whereas with the combination of cetuximab and carboplatin it was 17%. The dose of carboplatin administered in the above-mentioned study was AUC 2 weekly for 3 weeks over a 28-day cycle. Our patient population was similar to the Carey study with 44% of patients having received no prior treatment for metastatic disease. However, the combination of carboplatin and everolimus was able to achieve a much higher response rate (28% in our study) despite a lower dose of carboplatin, demonstrating synergy of the combination [29].

TNBC has been found to be sensitive to platinum-based treatment due to inherent genomic instability. In a series of 151 patients with metastatic TNBC, response rates with treatment using platinum combination therapy (cisplatin combined with either paclitaxel or vinorelbine) was higher than with other chemotherapy regimens (42% versus 23%). In another small series of eight patients with metastatic TNBC randomized to docetaxel/capecitabine combination versus docetaxel/cisplatin combination, the ORR was higher in the platinum-containing group that also translated into improved PFS and OS [30].

The mTOR inhibitor everolimus has been recently Food and Drug Administration-approved in combination with exemestane for the treatment of metastatic hormone receptor-positive breast cancer based on a significant improvement in PFS in the randomized phase III BOLERO-2 trial [24]. However, mTOR inhibitors have not been evaluated in a phase II or III study of TNBC. With TNBC there is high frequency of PTEN loss, which leads to mTOR activation. Moreover, it has been reported that mTOR activation confers resistance to platinum agents such as cisplatin, a phenomenon that is reversible by addition of an mTOR inhibitor such as everolimus [31].

In a phase I trial combining everolimus and carboplatin for metastatic breast cancer, patients were treated with weekly carboplatin (AUC 2) and everolimus at escalating doses of 2 · 5 mg/d, 5.0 mg/d, 7 · 5 mg/d or 10.0 mg/d, with 40% of patients receiving 10 mg/d. The study showed a CBR in 6/14 patients (43%). Of note, 4/6 patients treated with everolimus 10 mg/day had dose reductions due to grade-3 or -4 hematological toxicity or grade-4 fatigue. There was also a higher rate of mucositis (60%) observed, compared to only 4% in our study, likely due to the lower dose of everolimus. Grade-3 hematological toxicities observed in the phase I combination trial (thrombocytopenia, neutropenia and anemia) were comparable.

Strengths of our study are the prospective design and a clinical end point looking at the clinical benefit rate. While this study does not evaluate the contribution of everolimus on the observed efficacy, given the encouraging results, a randomized phase II trial of carboplatin alone versus carboplatin combined with everolimus in TNBC is in development. Furthermore, we observed an efficacy signal of everolimus in a patient who was progressing on single-agent carboplatin prior to treatment and achieved SD when everolimus was added. Similarly, a patient who had initially achieved PR with the combination maintained this response on single-agent everolimus for 8 · 3 months after carboplatin was discontinued secondary to neutropenia.

This trial has met the primary end point of demonstrating clinical benefit and describing the safety profile of everolimus and carboplatin combination in triple-negative metastatic breast cancer. Carboplatin at AUC 4 when combined with 5 mg daily everolimus is safe and efficacious.

## Conclusions

Everolimus-carboplatin combination was efficacious in metastatic TNBC. Dose-limiting hematological toxicity was observed when higher doses (AUC 5/6) of carboplatin were combined with everolimus. However, carboplatin AUC 4 was well tolerated in combination with everolimus, with continuing responses.

## Abbreviations

4E-BP1: 4E-binding protein 1; AC: anthracycline cyclophosphamide; ALT: alanine transaminase; AST: aspartate transaminase; AUC: area under curve; BRCA: breast cancer; CA 27/29: cancer antigen 27/29; CBR: clinical benefit rate; CEA: carcinoembryonic acid; CK 5/6: cytokeratin 5/6; CR: complete response; CT: computed tomography; DLCO: diffusion capacity of lung for carbon monoxide; EGFR: endothelial-derived growth factor; ER: estrogen receptor; FISH: fluorescent in-situ hybridization; HIF-1: hypoxia induced factor-1; HR: hazard ratio; IHC: immunohistochemistry; INR: international normalized ratio; IRB: Institutional Review Board; mTOR: mammalian target of Rapamycin; ORR: overall response rate; OS: overall survival; PD: progression of disease; PET: positron emission tomography; PFS: progression-free survival; PI3K: phosphotidyl inositol 3 kinase; PRes: partial response; PR: progesterone receptor; PT: prothrombin time; PTEN: phosphatase and tensin homolog; RECIST: response evaluation criteria in solid tumors; S6K1: S6 kinase 1; SD: stable disease; TNBC: triple-negative breast cancer; ULN: upper limit of normal; VEGF: vascular endothelial growth factor; WHO: World health organization.

## Competing interests

Funding for this work was provided by Novartis Pharmaceuticals Corporation.

## Authors’ contributions

JCS was responsible for data acquisition, data analysis, data interpretation, formatting data tables, writing and revision of the study manuscript. YN was involved in conception of study design, writing and revision of manuscript. SS, MV, MM, CO, JuS, RS and SF helped with data acquisition, original study design and conception, drafting the final manuscript and providing critical edits. VK, BJ and KJ helped with data acquisition, analysis, drafting the final manuscript and providing critical edits. JaS helped with data acquisition and manuscript revision. XL and JG helped with data interpretation, data analysis, construction of figures and tables and manuscript writing. SA helped with data acquisition, data analysis, manuscript revision and providing critical edits. AT was the principal investigator and was involved in study conception and design, writing of original protocol, data acquisition, data interpretation, drafting and revision of the final manuscript. All authors read and approved the final manuscript.

## Supplementary Material

Additional file 1: Table S1 Demographics table. Click here for file

Additional file 2: Table S2 Hematological toxicity table. This table shows hematological toxicity observed in patients with triple-negative metastatic breast cancer treated with everolimus and carboplatin combination. Hematological toxicity is further separated by severity that is, grade 1 to 2, grade 3 or grade 4. No cases of febrile neutropenia were observed. Click here for file

Additional file 3: Table S3 Non-hematological toxicity, which was related to the treatment regimen table. This table shows non-hematological toxicity observed in patients with triple-negative metastatic breast cancer treated with everolimus and carboplatin combination, which was directly related to the drug regimen. No grade-4-related toxicities were observed. Click here for file
