# Supplementary material for: Phase 2 trial of everolimus and carboplatin combination in patients with triple negative metastatic breast cancer
Source: Breast Cancer Res. 2014 Mar 31;16(2):R32. doi: 10.1186/bcr3634 (PMC4053575; doi:10.1186/bcr3634)
Supplement: Additional file 1: Table S1 — Demographics table. [file bcr3634-S1.docx]

| Additional file 1: Table S1 **PATIENT DEMOGRAPHICS** | | |
| --- | --- | --- |
| **VARIABLE** | **VALUE** | **PERCENTAGE** |
| Median age (years) | 58 |  |
|  |  |  |
| Performance status |  |  |
| 0 | 13 | 52% |
| 1 | 12 | 48% |
|  |  |  |
| Race |  |  |
| White non-Hispanic | 16 | 64% |
| Hispanic | 5 | 20% |
| African American | 2 | 8% |
| Other (Asian, Pacific Islander) | 2 | 8% |
|  |  |  |
| ER |  |  |
| <1% | 22 | 88% |
| 1-5% | 3 | 12% |
| 5-10% | 0 | 0 |
|  |  |  |
| PR |  |  |
| <1% | 23 | 92% |
| 1-5% | 1 | 4% |
| 5-10% | 1 | 4% |
|  |  |  |
| Site of metastases |  |  |
| Lymph nodes | 16 | 64% |
| Lung | 8 | 32% |
| Bone | 11 | 44% |
| Liver | 6 | 24% |
| Other (Peritoneum, chest wall, adrenal glands) | 2 | 8% |
|  |  |  |
| Prior Carboplatin | 2 | 8% |
|  |  |  |
| Prior regimens for metastatic disease |  | Median: 1 |
|  |  |  |
| 0 | 11 | 44% |
| 1 | 7 | 28% |
| 2 | 6 | 24% |
| 3 | 1 | 4% |
|  |  |  |
| Starting dose of Carboplatin |  |  |
| AUC 6 | 4 | 16% |
| AUC 5 | 3 | 12% |
| AUC 4 | 18 | 72% |
|  |  |  |
| Median number of treatment cycles received | 4 (range 1-19) |  |
