# Supplementary material for: Phase 2 trial of everolimus and carboplatin combination in patients with triple negative metastatic breast cancer
Source: Breast Cancer Res. 2014 Mar 31;16(2):R32. doi: 10.1186/bcr3634 (PMC4053575; doi:10.1186/bcr3634)
Supplement: Additional file 2: Table S2 — Hematological toxicity table. This table shows hematological toxicity observed in patients with triple-negative metastatic breast cancer treated with everolimus and carboplatin combination. Hematological toxicity is further separated by severity that is, grade 1 to 2, grade 3 or grade 4. No cases of febrile neutropenia were observed. [file bcr3634-S2.docx]

| Additional file 2: Table S2 **HEMATOLOGICAL TOXICITIES IN ALL PATIENTS** | | | |
| --- | --- | --- | --- |
| TOXICITY | GRADE 1-2 | GRADE 3 | GRADE 4 |
| **ANEMIA (n=25)** | 8 (32%) | 1 (4%) | 0 |
| Carboplatin AUC 5 or AUC 6 (n=7) | 4 (57%) | 0 | 0 |
| Carboplatin AUC 4 (n=18) | 4 (22%) | 1 (5%) | 0 |
| **THROMBOCYTOPENIA (n=25)** | 9 (36%) | 5 (20%) | 2 (8%) |
| Carboplatin AUC 5 or AUC 6 (n=7) | 1 (14%) | 3 (43%) | 2 (8%) |
| Carboplatin AUC 4 (n=18) | 8 (44%) | 2 (11%) | 0 |
| **LEUKOPENIA (n=25)** | 2 (8%) | 1 (4%) | 0 |
| Carboplatin AUC 5 or AUC 6 (n=7) | 2 (29%) | 1 (14%) | 0 |
| Carboplatin AUC 4 (n=18) | 0 | 0 | 0 |
| **NEUTROPENIA^A^**  **(n=25)** | 8 (32%) | 3 (12%)^ε^ | 0 |
| Carboplatin AUC 5 or AUC 6 (n=7) | 3 (43%) | 2 (29%) | 0 |
| Carboplatin AUC 4 (n=18) | 5 (27%) | 1 (5%) | 0 |
| ^A^ There were no cases of febrile neutropenia | | | |
