# Supplementary material for: Phase 2 trial of everolimus and carboplatin combination in patients with triple negative metastatic breast cancer
Source: Breast Cancer Res. 2014 Mar 31;16(2):R32. doi: 10.1186/bcr3634 (PMC4053575; doi:10.1186/bcr3634)
Supplement: Additional file 3: Table S3 — Non-hematological toxicity, which was related to the treatment regimen table. This table shows non-hematological toxicity observed in patients with triple-negative metastatic breast cancer treated with everolimus and carboplatin combination, which was directly related to the drug regimen. No grade-4-related toxicities were observed. [file bcr3634-S3.docx]

| Additional file 3: Table S3**: NON HEMATOLOGICAL TOXICITY RELATED TO TREATMENT REGIMEN** | | |
| --- | --- | --- |
| TOXICITY | GRADE 1-2 | GRADE 3 |
| **GASTROINTESTINAL** | | |
| NAUSEA | 10 (40%) | 1 (4%) |
| VOMITING | 5 (20%) | 1 (4%) |
| DIARRHEA | 1 (4%) | 0 |
| CONSTIPATION | 6 (24%) | 0 |
| ANOREXIA | 3 (12%) | 0 |
| ABDOMINAL PAIN | 1 (4%) | 0 |
| DEHYDRATION | 0 | 1 (4%) |
| ELEVATED LIVER ENZYMES | 2 (8%) | 0 |
| **CONSTITUTIONAL** | | |
| FATIGUE | 11 (44%) | 0 |
| FEVER | 1 (4%) | 0 |
| PRURITUS | 1 (4%) | 0 |
| **NEUROLOGICAL** | | |
| HEADACHE | 3 (12%) | 0 |
| NEUROPATHY | 2 (8%) | 0 |
| DIZZINESS/ VERTIGO | 1 (4%) | 0 |
| **SKIN/ MUCOUS MEMBRANES/EYE/ENT** | | |
| RASH | 6 (24%) | 0 |
| TASTE CHANGES | 2 (8%) | 0 |
| HOARSENESS | 1 (4%) | 0 |
| EPISTAXIS | 3 (12%) | 0 |
| DRY MOUTH | 1 (4%) | 0 |
| XEROPHTHALMIA | 1 (4%) | 0 |
| MUCOSITIS | 0 | 1 (4%) |
| **ALLERGIC** | | |
| HYPERSENSITIVITY | 0 | 1 (4%) |
| FACIAL FLUSHING | 1 (4%) | 0 |
| **BLEEDING** | | |
| BRUISING | 1 (4%) | 0 |
| VAGINAL SPOTTING | 1 (4%) | 0 |
| **RESPIRATORY** | | |
| COUGH | 1 (4%) | 0 |
| **RENAL** | | |
| PROTEINURIA | 1 (4%) | 0 |
